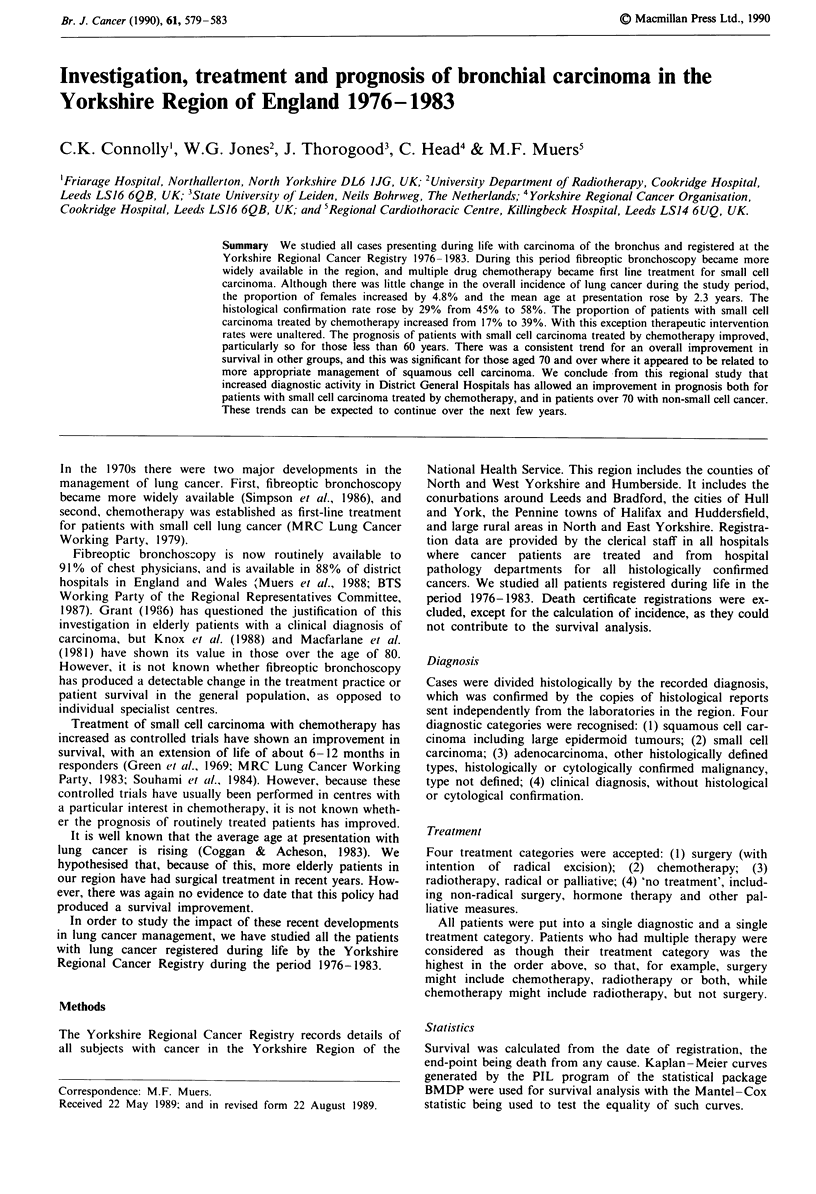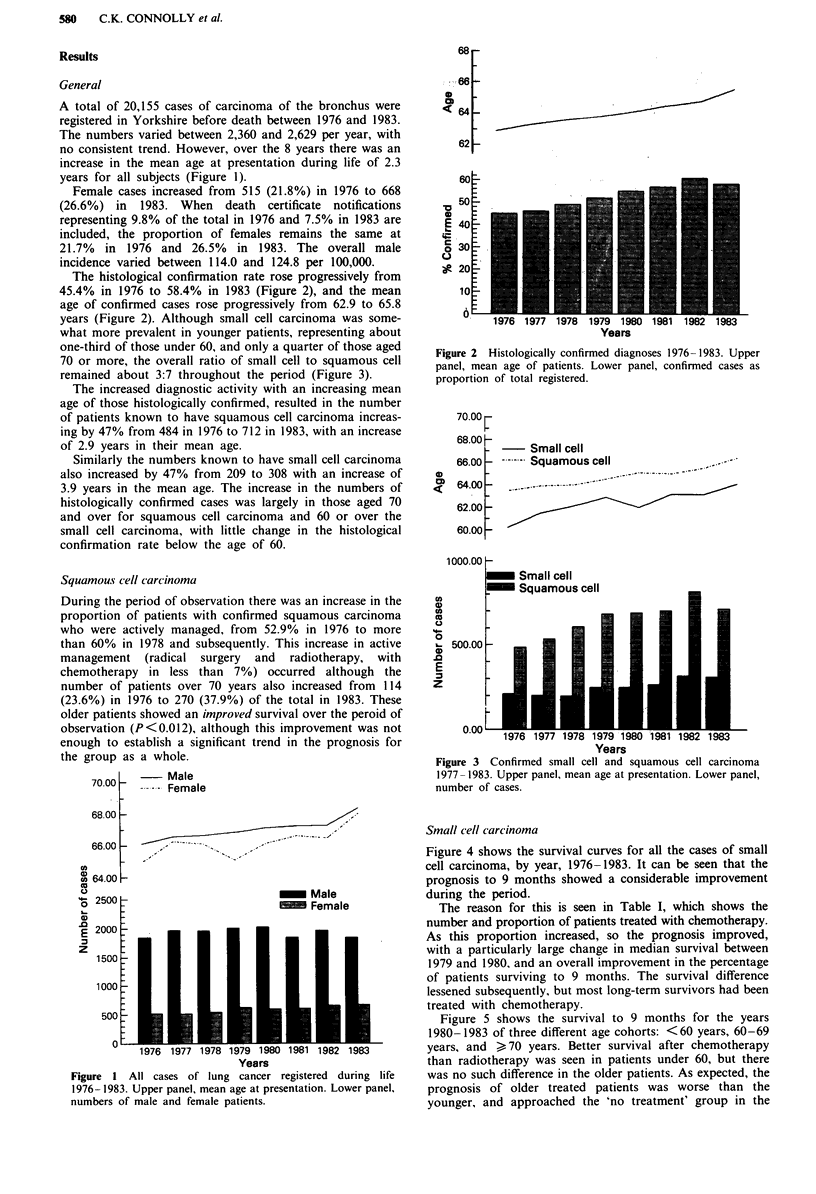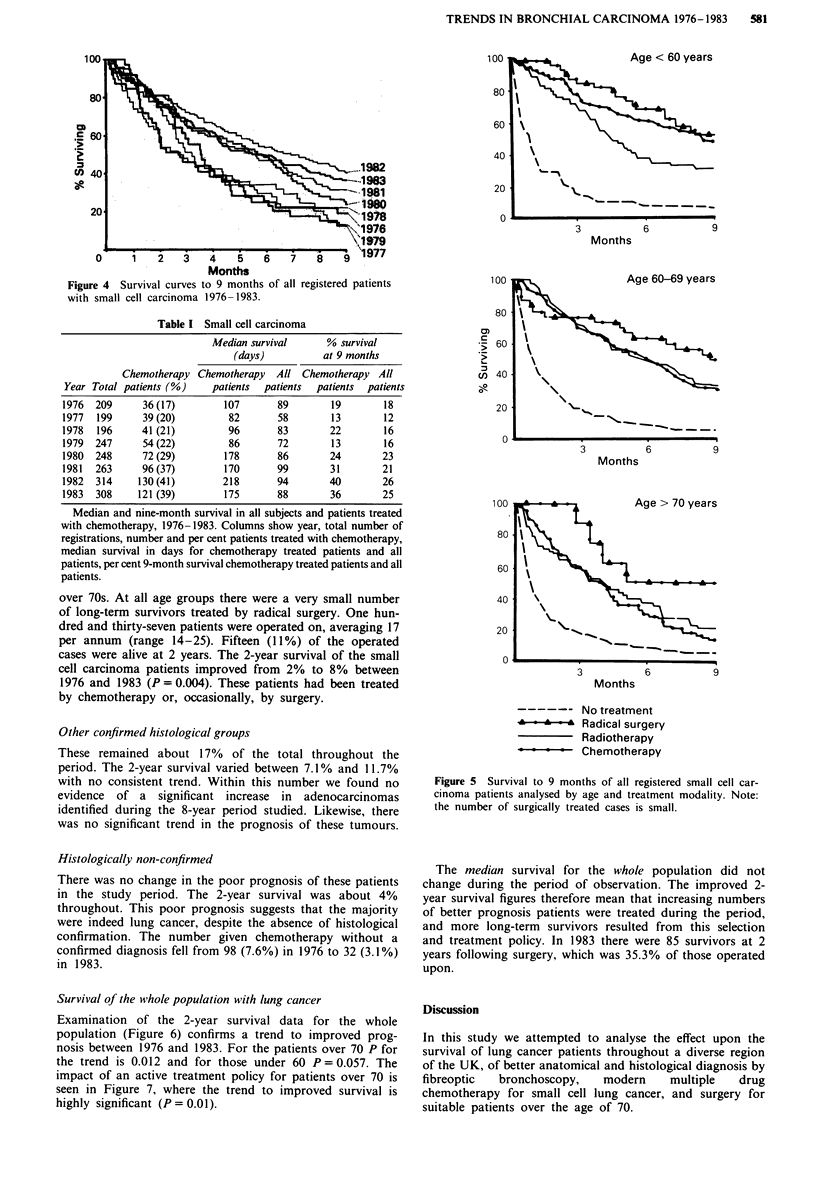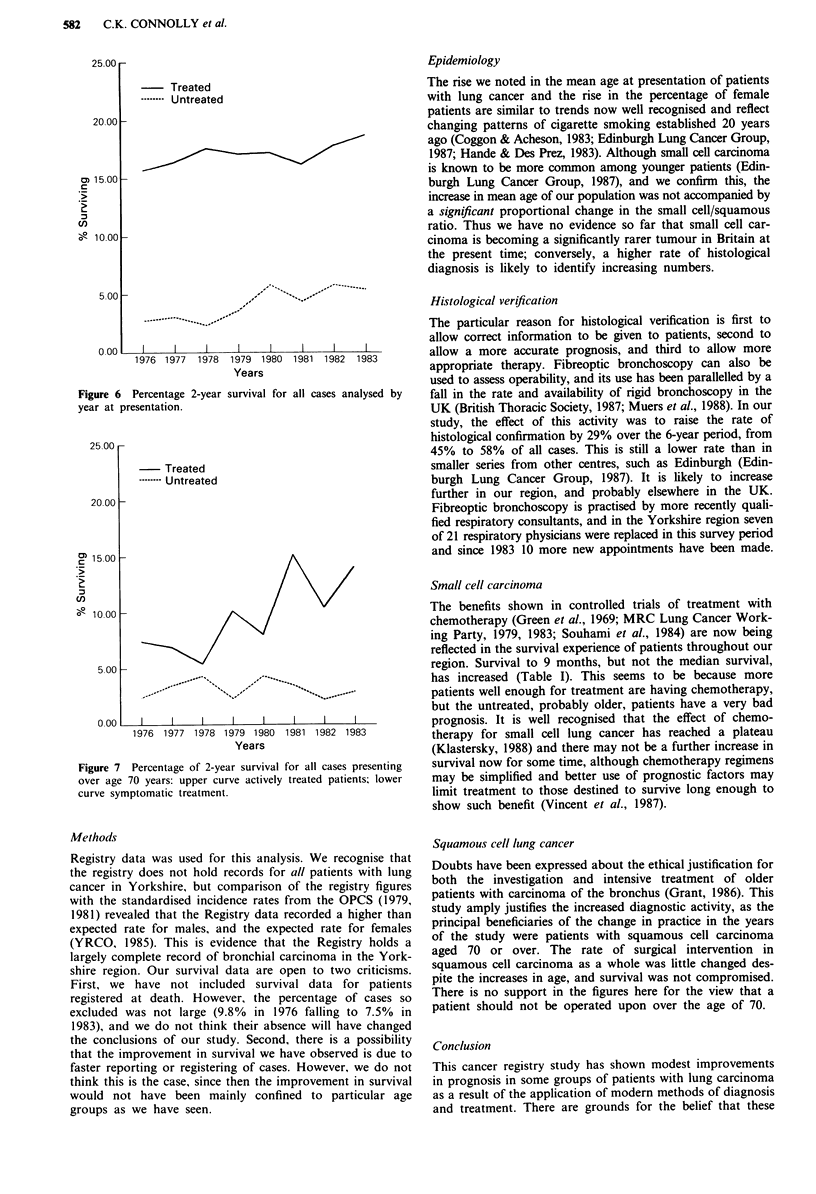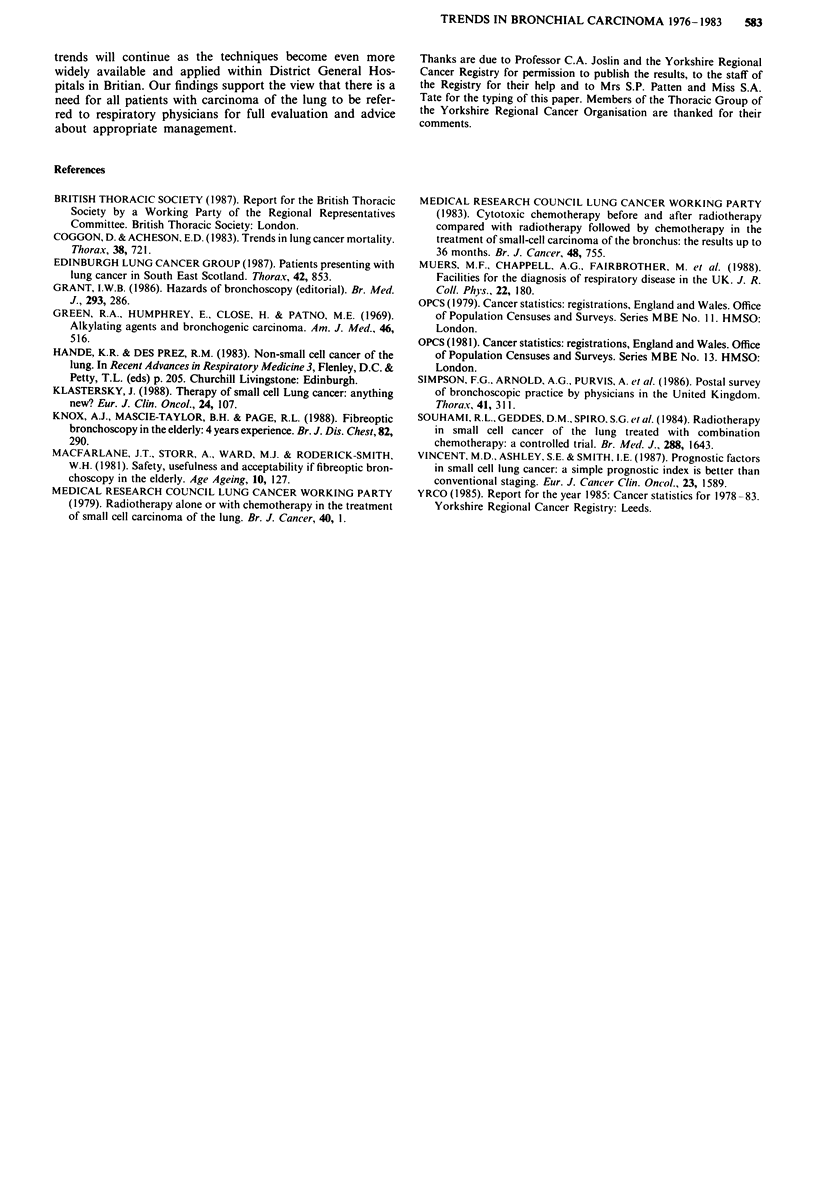# Investigation, treatment and prognosis of bronchial carcinoma in the Yorkshire Region of England 1976-1983.

**DOI:** 10.1038/bjc.1990.130

**Published:** 1990-04

**Authors:** C. K. Connolly, W. G. Jones, J. Thorogood, C. Head, M. F. Muers

**Affiliations:** Friarage Hospital, Northallerton, North Yorkshire, UK.

## Abstract

We studied all cases presenting during life with carcinoma of the bronchus and registered at the Yorkshire Regional Cancer Registry 1976-1983. During this period fibreoptic bronchoscopy became more widely available in the region, and multiple drug chemotherapy became first line treatment for small cell carcinoma. Although there was little change in the overall incidence of lung cancer during the study period, the proportion of females increased by 4.8% and the mean age at presentation rose by 2.3 years. The histological confirmation rate rose by 29% from 45% to 58%. The proportion of patients with small cell carcinoma treated by chemotherapy increased from 17% to 39%. With this exception therapeutic intervention rates were unaltered. The prognosis of patients with small cell carcinoma treated by chemotherapy improved, particularly so for those less than 60 years. There was a consistent trend for an overall improvement in survival in other groups, and this was significant for those aged 70 and over where it appeared to be related to more appropriate management of squamous cell carcinoma. We conclude from this regional study that increased diagnostic activity in District General Hospitals has allowed an improvement in prognosis both for patients with small cell carcinoma treated by chemotherapy, and in patients over 70 with non-small cell cancer. These trends can be expected to continue over the next few years.


Br. J. Cancer (1990), 61, 579-583 © Macmillan Press Ltd., 1990

### Investigation, treatment and prognosis of bronchial carcinoma in the Yorkshire Region of England 1976-1983

C.K. Connolly[1], W.G. Jones[2], J. Thorogood[3], C. Head[4] & M.F. Muers[5]

[1]*Friarage Hospital, Northallerton, North Yorkshire DL6 1JG, UK;* [2]*University Department of Radiotherapy, Cookridge Hospital, Leeds LS16 6QB, UK;* [3]*State University of Leiden, Neils Bohrweg, The Netherlands;* [4]*Yorkshire Regional Cancer Organisation, Cookridge Hospital, Leeds LS16 6QB, UK; and* [5]*Regional Cardiothoracic Centre, Killingbeck Hospital, Leeds LS14 6UQ, UK.*

**Summary** We studied all cases presenting during life with carcinoma of the bronchus and registered at the Yorkshire Regional Cancer Registry 1976-1983. During this period fibreoptic bronchoscopy became more widely available in the region, and multiple drug chemotherapy became first line treatment for small cell carcinoma. Although there was little change in the overall incidence of lung cancer during the study period, the proportion of females increased by 4.8% and the mean age at presentation rose by 2.3 years. The histological confirmation rate rose by 29% from 45% to 58%. The proportion of patients with small cell carcinoma treated by chemotherapy increased from 17% to 39%. With this exception therapeutic intervention rates were unaltered. The prognosis of patients with small cell carcinoma treated by chemotherapy improved, particularly so for those less than 60 years. There was a consistent trend for an overall improvement in survival in other groups, and this was significant for those aged 70 and over where it appeared to be related to more appropriate management of squamous cell carcinoma. We conclude from this regional study that increased diagnostic activity in District General Hospitals has allowed an improvement in prognosis both for patients with small cell carcinoma treated by chemotherapy, and in patients over 70 with non-small cell cancer. These trends can be expected to continue over the next few years.

In the 1970s there were two major developments in the management of lung cancer. First, fibreoptic bronchoscopy became more widely available (Simpson *et al.*, 1986), and second, chemotherapy was established as first-line treatment for patients with small cell lung cancer (MRC Lung Cancer Working Party, 1979).

Fibreoptic bronchoscopy is now routinely available to 91% of chest physicians, and is available in 88% of district hospitals in England and Wales (Muers *et al.*, 1988; BTS Working Party of the Regional Representatives Committee, 1987). Grant (1986) has questioned the justification of this investigation in elderly patients with a clinical diagnosis of carcinoma, but Knox *et al.* (1988) and Macfarlane *et al.* (1981) have shown its value in those over the age of 80. However, it is not known whether fibreoptic bronchoscopy has produced a detectable change in the treatment practice or patient survival in the general population, as opposed to individual specialist centres.

Treatment of small cell carcinoma with chemotherapy has increased as controlled trials have shown an improvement in survival, with an extension of life of about 6-12 months in responders (Green *et al.*, 1969; MRC Lung Cancer Working Party, 1983; Souhami *et al.*, 1984). However, because these controlled trials have usually been performed in centres with a particular interest in chemotherapy, it is not known whether the prognosis of routinely treated patients has improved.

It is well known that the average age at presentation with lung cancer is rising (Coggan & Acheson, 1983). We hypothesised that, because of this, more elderly patients in our region have had surgical treatment in recent years. However, there was again no evidence to date that this policy had produced a survival improvement.

In order to study the impact of these recent developments in lung cancer management, we have studied all the patients with lung cancer registered during life by the Yorkshire Regional Cancer Registry during the period 1976-1983.

### Methods

The Yorkshire Regional Cancer Registry records details of all subjects with cancer in the Yorkshire Region of the National Health Service. This region includes the counties of North and West Yorkshire and Humberside. It includes the conurbations around Leeds and Bradford, the cities of Hull and York, the Pennine towns of Halifax and Huddersfield, and large rural areas in North and East Yorkshire. Registration data are provided by the clerical staff in all hospitals where cancer patients are treated and from hospital pathology departments for all histologically confirmed cancers. We studied all patients registered during life in the period 1976-1983. Death certificate registrations were excluded, except for the calculation of incidence, as they could not contribute to the survival analysis.

### *Diagnosis*

Cases were divided histologically by the recorded diagnosis, which was confirmed by the copies of histological reports sent independently from the laboratories in the region. Four diagnostic categories were recognised: (1) squamous cell carcinoma including large epidermoid tumours; (2) small cell carcinoma; (3) adenocarcinoma, other histologically defined types, histologically or cytologically confirmed malignancy, type not defined; (4) clinical diagnosis, without histological or cytological confirmation.

### *Treatment*

Four treatment categories were accepted: (1) surgery (with intention of radical excision); (2) chemotherapy; (3) radiotherapy, radical or palliative; (4) 'no treatment', including non-radical surgery, hormone therapy and other palliative measures.

All patients were put into a single diagnostic and a single treatment category. Patients who had multiple therapy were considered as though their treatment category was the highest in the order above, so that, for example, surgery might include chemotherapy, radiotherapy or both, while chemotherapy might include radiotherapy, but not surgery.

### *Statistics*

Survival was calculated from the date of registration, the end-point being death from any cause. Kaplan-Meier curves generated by the PIL program of the statistical package BMDP were used for survival analysis with the Mantel-Cox statistic being used to test the equality of such curves.

Correspondence: M.F. Muers.
Received 22 May 1989; and in revised form 22 August 1989.

### Results

### *General*

A total of 20,155 cases of carcinoma of the bronchus were registered in Yorkshire before death between 1976 and 1983. The numbers varied between 2,360 and 2,629 per year, with no consistent trend. However, over the 8 years there was an increase in the mean age at presentation during life of 2.3 years for all subjects (Figure 1).

Female cases increased from 515 (21.8%) in 1976 to 668 (26.6%) in 1983. When death certificate notifications representing 9.8% of the total in 1976 and 7.5% in 1983 are included, the proportion of females remains the same at 21.7% in 1976 and 26.5% in 1983. The overall male incidence varied between 114.0 and 124.8 per 100,000.

The histological confirmation rate rose progressively from 45.4% in 1976 to 58.4% in 1983 (Figure 2), and the mean age of confirmed cases rose progressively from 62.9 to 65.8 years (Figure 2). Although small cell carcinoma was somewhat more prevalent in younger patients, representing about one-third of those under 60, and only a quarter of those aged 70 or more, the overall ratio of small cell to squamous cell remained about 3:7 throughout the period (Figure 3).

The increased diagnostic activity with an increasing mean age of those histologically confirmed, resulted in the number of patients known to have squamous cell carcinoma increasing by 47% from 484 in 1976 to 712 in 1983, with an increase of 2.9 years in their mean age.

Similarly the numbers known to have small cell carcinoma also increased by 47% from 209 to 308 with an increase of 3.9 years in the mean age. The increase in the numbers of histologically confirmed cases was largely in those aged 70 and over for squamous cell carcinoma and 60 or over the small cell carcinoma, with little change in the histological confirmation rate below the age of 60.

### *Squamous cell carcinoma*

During the period of observation there was an increase in the proportion of patients with confirmed squamous carcinoma who were actively managed, from 52.9% in 1976 to more than 60% in 1978 and subsequently. This increase in active management (radical surgery and radiotherapy, with chemotherapy in less than 7%) occurred although the number of patients over 70 years also increased from 114 (23.6%) in 1976 to 270 (37.9%) of the total in 1983. These older patients showed an *improved* survival over the peroid of observation ($P < 0.012$), although this improvement was not enough to establish a significant trend in the prognosis for the group as a whole.

**Figure 1** All cases of lung cancer registered during life 1976–1983. Upper panel, mean age at presentation. Lower panel, numbers of male and female patients.

**Figure 2** Histologically confirmed diagnoses 1976–1983. Upper panel, mean age of patients. Lower panel, confirmed cases as proportion of total registered.

**Figure 3** Confirmed small cell and squamous cell carcinoma 1977–1983. Upper panel, mean age at presentation. Lower panel, number of cases.

### *Small cell carcinoma*

Figure 4 shows the survival curves for all the cases of small cell carcinoma, by year, 1976–1983. It can be seen that the prognosis to 9 months showed a considerable improvement during the period.

The reason for this is seen in Table I, which shows the number and proportion of patients treated with chemotherapy. As this proportion increased, so the prognosis improved, with a particularly large change in median survival between 1979 and 1980, and an overall improvement in the percentage of patients surviving to 9 months. The survival difference lessened subsequently, but most long-term survivors had been treated with chemotherapy.

Figure 5 shows the survival to 9 months for the years 1980–1983 of three different age cohorts: <60 years, 60–69 years, and ≥70 years. Better survival after chemotherapy than radiotherapy was seen in patients under 60, but there was no such difference in the older patients. As expected, the prognosis of older treated patients was worse than the younger, and approached the 'no treatment' group in the

**Figure 4** Survival curves to 9 months of all registered patients with small cell carcinoma 1976–1983.

**Table I** Small cell carcinoma

| Year | Total | Chemotherapy patients (%) | Median survival (days) Chemotherapy patients | All patients | % survival at 9 months Chemotherapy patients | All patients |
|---|---|---|---|---|---|---|
| 1976 | 209 | 36 (17) | 107 | 89 | 19 | 18 |
| 1977 | 199 | 39 (20) | 82 | 58 | 13 | 12 |
| 1978 | 196 | 41 (21) | 96 | 83 | 22 | 16 |
| 1979 | 247 | 54 (22) | 86 | 72 | 13 | 16 |
| 1980 | 248 | 72 (29) | 178 | 86 | 24 | 23 |
| 1981 | 263 | 96 (37) | 170 | 99 | 31 | 21 |
| 1982 | 314 | 130 (41) | 218 | 94 | 40 | 26 |
| 1983 | 308 | 121 (39) | 175 | 88 | 36 | 25 |

Median and nine-month survival in all subjects and patients treated with chemotherapy, 1976–1983. Columns show year, total number of registrations, number and per cent patients treated with chemotherapy, median survival in days for chemotherapy treated patients and all patients, per cent 9-month survival chemotherapy treated patients and all patients.

over 70s. At all age groups there were a very small number of long-term survivors treated by radical surgery. One hundred and thirty-seven patients were operated on, averaging 17 per annum (range 14–25). Fifteen (11%) of the operated cases were alive at 2 years. The 2-year survival of the small cell carcinoma patients improved from 2% to 8% between 1976 and 1983 ($P = 0.004$). These patients had been treated by chemotherapy or, occasionally, by surgery.

### *Other confirmed histological groups*

These remained about 17% of the total throughout the period. The 2-year survival varied between 7.1% and 11.7% with no consistent trend. Within this number we found no evidence of a significant increase in adenocarcinomas identified during the 8-year period studied. Likewise, there was no significant trend in the prognosis of these tumours.

### *Histologically non-confirmed*

There was no change in the poor prognosis of these patients in the study period. The 2-year survival was about 4% throughout. This poor prognosis suggests that the majority were indeed lung cancer, despite the absence of histological confirmation. The number given chemotherapy without a confirmed diagnosis fell from 98 (7.6%) in 1976 to 32 (3.1%) in 1983.

### *Survival of the whole population with lung cancer*

Examination of the 2-year survival data for the whole population (Figure 6) confirms a trend to improved prognosis between 1976 and 1983. For the patients over 70 $P$ for the trend is 0.012 and for those under 60 $P = 0.057$. The impact of an active treatment policy for patients over 70 is seen in Figure 7, where the trend to improved survival is highly significant ($P = 0.01$).

**Figure 5** Survival to 9 months of all registered small cell carcinoma patients analysed by age and treatment modality. Note: the number of surgically treated cases is small.

The *median* survival for the *whole* population did not change during the period of observation. The improved 2-year survival figures therefore mean that increasing numbers of better prognosis patients were treated during the period, and more long-term survivors resulted from this selection and treatment policy. In 1983 there were 85 survivors at 2 years following surgery, which was 35.3% of those operated upon.

### Discussion

In this study we attempted to analyse the effect upon the survival of lung cancer patients throughout a diverse region of the UK, of better anatomical and histological diagnosis by fibreoptic bronchoscopy, modern multiple drug chemotherapy for small cell lung cancer, and surgery for suitable patients over the age of 70.

**Figure 6** Percentage 2-year survival for all cases analysed by year at presentation.

**Figure 7** Percentage of 2-year survival for all cases presenting over age 70 years: upper curve actively treated patients; lower curve symptomatic treatment.

### *Methods*

Registry data was used for this analysis. We recognise that the registry does not hold records for *all* patients with lung cancer in Yorkshire, but comparison of the registry figures with the standardised incidence rates from the OPCS (1979, 1981) revealed that the Registry data recorded a higher than expected rate for males, and the expected rate for females (YRCO, 1985). This is evidence that the Registry holds a largely complete record of bronchial carcinoma in the Yorkshire region. Our survival data are open to two criticisms. First, we have not included survival data for patients registered at death. However, the percentage of cases so excluded was not large (9.8% in 1976 falling to 7.5% in 1983), and we do not think their absence will have changed the conclusions of our study. Second, there is a possibility that the improvement in survival we have observed is due to faster reporting or registering of cases. However, we do not think this is the case, since then the improvement in survival would not have been mainly confined to particular age groups as we have seen.

### *Epidemiology*

The rise we noted in the mean age at presentation of patients with lung cancer and the rise in the percentage of female patients are similar to trends now well recognised and reflect changing patterns of cigarette smoking established 20 years ago (Coggon & Acheson, 1983; Edinburgh Lung Cancer Group, 1987; Hande & Des Prez, 1983). Although small cell carcinoma is known to be more common among younger patients (Edinburgh Lung Cancer Group, 1987), and we confirm this, the increase in mean age of our population was not accompanied by a *significant* proportional change in the small cell/squamous ratio. Thus we have no evidence so far that small cell carcinoma is becoming a significantly rarer tumour in Britain at the present time; conversely, a higher rate of histological diagnosis is likely to identify increasing numbers.

### *Histological verification*

The particular reason for histological verification is first to allow correct information to be given to patients, second to allow a more accurate prognosis, and third to allow more appropriate therapy. Fibreoptic bronchoscopy can also be used to assess operability, and its use has been parallelled by a fall in the rate and availability of rigid bronchoscopy in the UK (British Thoracic Society, 1987; Muers *et al.*, 1988). In our study, the effect of this activity was to raise the rate of histological confirmation by 29% over the 6-year period, from 45% to 58% of all cases. This is still a lower rate than in smaller series from other centres, such as Edinburgh (Edinburgh Lung Cancer Group, 1987). It is likely to increase further in our region, and probably elsewhere in the UK. Fibreoptic bronchoscopy is practised by more recently qualified respiratory consultants, and in the Yorkshire region seven of 21 respiratory physicians were replaced in this survey period and since 1983 10 more new appointments have been made.

### *Small cell carcinoma*

The benefits shown in controlled trials of treatment with chemotherapy (Green *et al.*, 1969; MRC Lung Cancer Working Party, 1979, 1983; Souhami *et al.*, 1984) are now being reflected in the survival experience of patients throughout our region. Survival to 9 months, but not the median survival, has increased (Table I). This seems to be because more patients well enough for treatment are having chemotherapy, but the untreated, probably older, patients have a very bad prognosis. It is well recognised that the effect of chemotherapy for small cell lung cancer has reached a plateau (Klastersky, 1988) and there may not be a further increase in survival now for some time, although chemotherapy regimens may be simplified and better use of prognostic factors may limit treatment to those destined to survive long enough to show such benefit (Vincent *et al.*, 1987).

### *Squamous cell lung cancer*

Doubts have been expressed about the ethical justification for both the investigation and intensive treatment of older patients with carcinoma of the bronchus (Grant, 1986). This study amply justifies the increased diagnostic activity, as the principal beneficiaries of the change in practice in the years of the study were patients with squamous cell carcinoma aged 70 or over. The rate of surgical intervention in squamous cell carcinoma as a whole was little changed despite the increases in age, and survival was not compromised. There is no support in the figures here for the view that a patient should not be operated upon over the age of 70.

### *Conclusion*

This cancer registry study has shown modest improvements in prognosis in some groups of patients with lung carcinoma as a result of the application of modern methods of diagnosis and treatment. There are grounds for the belief that these trends will continue as the techniques become even more widely available and applied within District General Hospitals in Britian. Our findings support the view that there is a need for all patients with carcinoma of the lung to be referred to respiratory physicians for full evaluation and advice about appropriate management.

Thanks are due to Professor C.A. Joslin and the Yorkshire Regional Cancer Registry for permission to publish the results, to the staff of the Registry for their help and to Mrs S.P. Patten and Miss S.A. Tate for the typing of this paper. Members of the Thoracic Group of the Yorkshire Regional Cancer Organisation are thanked for their comments.

**References**

BRITISH THORACIC SOCIETY (1987). Report for the British Thoracic Society by a Working Party of the Regional Representatives Committee. British Thoracic Society: London.

COGGON, D. & ACHESON, E.D. (1983). Trends in lung cancer mortality. *Thorax*, **38**, 721.

EDINBURGH LUNG CANCER GROUP (1987). Patients presenting with lung cancer in South East Scotland. *Thorax*, **42**, 853.

GRANT, I.W.B. (1986). Hazards of bronchoscopy (editorial). *Br. Med. J.*, **293**, 286.

GREEN, R.A., HUMPHREY, E., CLOSE, H. & PATNO, M.E. (1969). Alkylating agents and bronchogenic carcinoma. *Am. J. Med.*, **46**, 516.

HANDE, K.R. & DES PREZ, R.M. (1983). Non-small cell cancer of the lung. In *Recent Advances in Respiratory Medicine 3*, Flenley, D.C. & Petty, T.L. (eds) p. 205. Churchill Livingstone: Edinburgh.

KLASTERSKY, J. (1988). Therapy of small cell Lung cancer: anything new? *Eur. J. Clin. Oncol.*, **24**, 107.

KNOX, A.J., MASCIE-TAYLOR, B.H. & PAGE, R.L. (1988). Fibreoptic bronchoscopy in the elderly: 4 years experience. *Br. J. Dis. Chest*, **82**, 290.

MACFARLANE, J.T., STORR, A., WARD, M.J. & RODERICK-SMITH, W.H. (1981). Safety, usefulness and acceptability if fibreoptic bronchoscopy in the elderly. *Age Ageing*, **10**, 127.

MEDICAL RESEARCH COUNCIL LUNG CANCER WORKING PARTY (1979). Radiotherapy alone or with chemotherapy in the treatment of small cell carcinoma of the lung. *Br. J. Cancer*, **40**, 1.

MEDICAL RESEARCH COUNCIL LUNG CANCER WORKING PARTY (1983). Cytotoxic chemotherapy before and after radiotherapy compared with radiotherapy followed by chemotherapy in the treatment of small-cell carcinoma of the bronchus: the results up to 36 months. *Br. J. Cancer*, **48**, 755.

MUERS, M.F., CHAPPELL, A.G., FAIRBROTHER, M. *et al.* (1988). Facilities for the diagnosis of respiratory disease in the UK. *J. R. Coll. Phys.*, **22**, 180.

OPCS (1979). Cancer statistics: registrations, England and Wales. Office of Population Censuses and Surveys. Series MBE No. 11. HMSO: London.

OPCS (1981). Cancer statistics: registrations, England and Wales. Office of Population Censuses and Surveys. Series MBE No. 13. HMSO: London.

SIMPSON, F.G., ARNOLD, A.G., PURVIS, A. *et al.* (1986). Postal survey of bronchoscopic practice by physicians in the United Kingdom. *Thorax*, **41**, 311.

SOUHAMI, R.L., GEDDES, D.M., SPIRO, S.G. *et al.* (1984). Radiotherapy in small cell cancer of the lung treated with combination chemotherapy: a controlled trial. *Br. Med. J.*, **288**, 1643.

VINCENT, M.D., ASHLEY, S.E. & SMITH, I.E. (1987). Prognostic factors in small cell lung cancer: a simple prognostic index is better than conventional staging. *Eur. J. Cancer Clin. Oncol.*, **23**, 1589.

YRCO (1985). Report for the year 1985: Cancer statistics for 1978–83. Yorkshire Regional Cancer Registry: Leeds.